# In Silico Prediction of Alkaline Phosphatase Interaction with the Natural Inhibitory 5-Azaindoles Guitarrin C and D

**DOI:** 10.3390/molecules29235701

**Published:** 2024-12-03

**Authors:** Aleksandra Seitkalieva, Yulia Noskova, Marina Isaeva, Alla Guzii, Tatyana N. Makarieva, Sergey Fedorov, Larissa Balabanova

**Affiliations:** 1 G.B. Elyakov Pacific Institute of Bioorganic Chemistry, Far Eastern Branch, Russian Academy of Sciences, Prospect 100-Letya Vladivostoka 152, 690022 Vladivostok, Russia; sasha0788@inbox.ru (A.S.); noskovaiulia@yandex.ru (Y.N.); issaeva@gmail.com (M.I.); gagry@rambler.ru (A.G.); makarieva@piboc.dvo.ru (T.N.M.); fedorov@piboc.dvo.ru (S.F.); 2 Youth Research Laboratory of Recombinant DNA Technologies, Advanced Engineering School, Institute of Biotechnology, Bioengineering and Food Systems, Far Eastern Federal University, 10 Ajax Bay, Russky Island, 690922 Vladivostok, Russia

**Keywords:** alkaline phosphatase, *Cobetia amphilecti*, marine sponge 5-azaindoles, guitarrins, non-competitive inhibitor, molecular docking

## Abstract

The natural 5-azaindoles, marine sponge guitarrin C and D, were observed to exert inhibitory activity against a highly active alkaline phosphatase (ALP) CmAP of the PhoA family from the marine bacterium *Cobetia amphilecti*, with IC_50_ values of 8.5 and 110 µM, respectively. The superimposition of CmAP complexes with *p*-nitrophenyl phosphate (*p*NPP), a commonly used chromogenic aryl substrate for ALP, and the inhibitory guitarrins C, D, and the non-inhibitory guitarrins A, B, and E revealed that the presence of a carboxyl group at C6 together with a hydroxyl group at C8 is a prerequisite for the inhibitory effect of 5-azaindoles on ALP activity. The 10-fold more active guitarrin C could compete with *p*NPP for binding sites in the ALP active site due to similarities in size, three-dimensional structure, and the orientation of the COOH group along the phosphate group. However, the inhibition of CmAP and calf intestinal ALP (CIAP) by guitarrin C was observed to occur via a non-competitive mode of action, as evidenced by a twofold decrease in V_max_ and an unchanged K_m_. In contrast, the kinetic model with guitarrin D, with an additional OH group at C7, reflected a mixed type of inhibition, with a decrease in both values. The sensitivity of CIAP to guitarrins C and D was shown to be slightly lower than that of CmAP, with IC_50_ values of 195 and 230 µM, respectively. Nevertheless, these findings prompted the prediction of complexes of human ALP isoenzymes with guitarrins C and D.

## 1. Introduction

Alkaline phosphatase (ALP) isoenzymes are of significant physiological and therapeutic interest due to their high expression in a number of severe metabolic disorders and diseases, including bone resorption (osteoporosis, Paget’s disease), abnormal calcification (soft tissues, arteries, brain), congestive heart failure, liver and kidney dysfunction, sepsis, and malignant obstruction [1,2,3,4,5,6,7,8,9]. Together with specific ecto-enzymes, such as nucleoside triphosphate diphosphohydrolase CD39 (ENTPD), nucleotide pyrophosphatase/phosphodiesterase (eNPP1), and ecto-5′-nucleotidase CD73 (e5′-NT), the non-specific phosphomonoesterase ALP plays an essential role in cellular regulation [6]. It is responsible for the hydrolysis of extracellular signaling nucleotides to nucleosides, in particular adenosine, and for the control of the availability of these signaling molecules at their respective receptors, which trigger different cellular processes [6,7,8]. It has been demonstrated that aberrant expression of the tissue-nonspecific (TNAP) and intestinal (IAP) isoenzymes is associated with a number of different cancers, including esophageal, breast, prostate, colon, ovarian, and liver cancer [9]. Thus, placental alkaline phosphatase (PLAP) was found to play a role in the formation of calcifications in the human breast cancer MDA-MB-231 cells through the PI3K-Akt signaling pathways, which is a process analogous to osteoblast differentiation [10]. Furthermore, ALP isoenzymes have been identified as key players in a complex anti-inflammatory mechanism due to their substrate non-specificity towards the terminal phosphate of cell-free signaling purines and pyrimidines, the cofactor nicotinamide adenine dinucleotide phosphate (NADPH) and its precursor nicotinamide mononucleotide (NMN) [6,8]. The aforementioned processes are also involved in the metabolism of DNA, RNA, and transmembrane phosphomonoesters, such as scavenger receptor fatty acid translocase B2/CB36, phosphoinositides, and protein kinases and their allosterically phosphorylated dual-specificity phosphatases [6,7,8,10,11,12,13].

**Figure 1:**
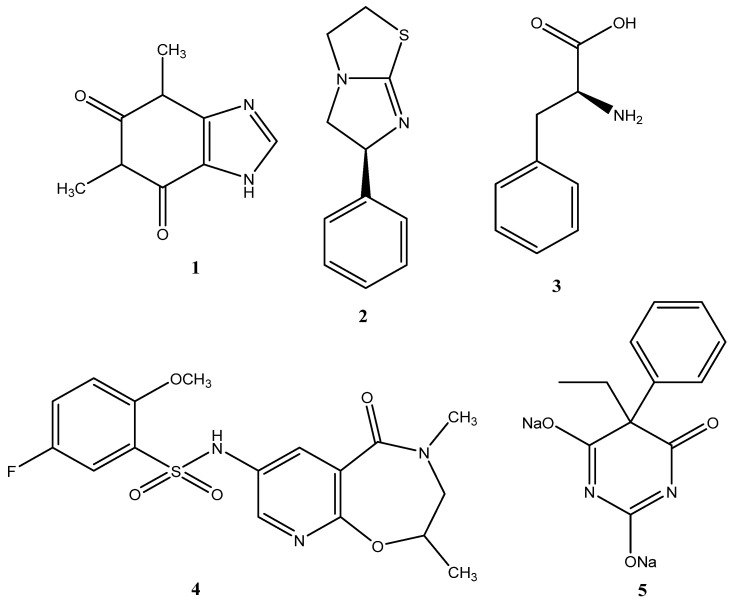
Conventional and newly discovered ALP inhibitors: theophylline (**1**); levamisole (**2**); L-phenylalanine (**3**); pyrido-oxazinone derivative DS-1211 (**4**); phenobarbital (**5**).

The identification of effective natural or synthetic inhibitors of ALP represents a promising approach to drug discovery, particularly in the context of aberrant activity associated with TNAP, IAP and PLAP isoenzymes. Furthermore, this approach may facilitate a deeper understanding of the role of ALP in cellular processes, potentially offering new avenues for modulating these processes through the use of these inhibitors [3,4,14,15,16,17,18,19]. It has been shown that heterocyclic compounds, a purine derivative, theophylline (IC_50_ = 47 µM; K_i_ = 91 µM) (**1**), and an imidazothiazole derivative, levamisole (IC_50_ = 19 µM) (**2**), as well as L-phenylalanine (IC_50_ = 80 µM) (**3**), are well-known inhibitors of ALP and are used as standard inhibitory ligands [17,18,19] (Figure 1).

Recently, however, several groups have identified a number of pyrazole, benzo[b]thiophene, 1,2,4-triazole and 1,3,4-thiadiazole, 3,3′-carbonyl-bis(chromones), isonicotinohydrazide, sulfonamide, bisphosphonates, polyphenols, and quinoline derivatives, which are potent and selective inhibitors of ALP isoenzymes [5,9,18]. For example, the first clinical trial of a TNAP-specific inhibitor, DS-1211, a pyrido-oxazinone derivative (**4**), for the treatment of ectopic calcification has recently been completed [5]. A commonly used anticonvulsant barbiturate, phenobarbital-5-ethyl-5-phenyl-2,4,6(1H,3H,5H)-pyrimidinetrione (**5**), has been shown to be an effective drug against the ALP target [9] (Figure 1). It is important to note, however, that many of the ALP-specific ligands that have been identified may be involved in undesirable vital pathways, which could result in adverse effects. For example, the common ALP inhibitors, including vanadate, pervanadate, and okadaic acid, have been demonstrated to impact cell viability, functionality, growth, and uptake by inducing alterations in the redox state of mitochondria [20]. Given the lack of clarity surrounding the mechanisms of many pathophysiological conditions associated with increased plasma ALP activity, the study of specific and non-specific ALP binding is a valuable avenue for elucidating the ALP-specific role in metabolic pathways [2,4,13,14,21].

The interactions of small organic molecules with ALP proteins are currently being investigated through molecular docking in order to predict their mechanism of action, binding efficiency, and inactivation potency. Consequently, a series of novel tricyclic coumarin sulphonate esters, benzocoumarin thiazole–azomethines, bisthioureas of pimelic and 4-methylsalicylic acids, and quinoline-4-carboxylic acid and pyrazolo-oxothiazolidine derivatives have been predicted in silico to exert competitive, non-competitive, or uncompetitive inhibitory effects on human ALP isozymes [14,16,19,22]. To achieve this, the structures of TNAP and IAP were constructed using molecular modelling techniques based on the PLAP crystal structure, which was determined at 1.8 A resolution and has long been the only available experimental model for human ALPs [23,24,25]. However, the crystal structures of TNAP and its dysfunctional mutants responsible for hypophosphatasia, a metabolic bone disease that manifests as developmental abnormalities in bone and dental tissues, have been recently determined [26].

In the context of microorganisms, both inorganic and organic ALP inhibitors are valuable tools for investigating the mechanisms of enzymatic catalysis and the biogeochemical roles of these processes in the environment. Such applications include the regulation of microbiomes, induced mineralization in biofilms, and the remediation of heavy metals and organic contaminants [27,28,29,30,31,32,33]. The notable inhibitory activity of guitarrins, the inaugural structurally characterized natural 5-azaindoles extracted from the Northwest Pacific marine sponge *Guitarra fimbriata*, against the highly active alkaline phosphatase CmAP from the marine bacterium *C. amphilecti* KMM 296, has recently been documented [33,34]. In comparison with the chelator ethylenediamine tetraacetic acid (EDTA), the compounds guitarrins C and D exhibited greater activity against the enzyme, with a 1000-fold and 100-fold higher affinity, respectively [33]. Previously, azaindole derivatives have been evaluated as potential kinase inhibitors based on a number of different kinase targets, including adaptor-associated kinase 1 (AAK1), anaplastic lymphocyte kinase (ALK), AXL, cell division cycle 7 (Cdc7), cyclin-dependent kinases (CDKs), dual-specificity tyrosine (Y)-phosphorylation-regulated kinase 1A (DYRK1A), and so forth. It has been observed that azaindole heterocycles interact with the ATP binding sites of the fibroblast growth factor receptor 4 (FGFR4), phosphatidylinositol 3-kinase (PI3K), and other targets. This interaction is attributed to the ability of two nitrogen atoms from the heterocycles to interact with the aforementioned sites, as evidenced by previous studies [34,35].

This study provides data on the ligand structural features responsible for the inhibitory activity of the marine sponge-derived 5-azaindoles against the *C. amphilecti* KMM 296 alkaline phosphatase CmAP through kinetics and molecular docking. Experimental kinetics were applied to both CmAP and calf intestinal alkaline phosphatase (CIAP) to compare the inhibitory activity of guitarrins C and D and their mode of inhibition. In the absence of the solved crystal structure of CIAP, the amino acid residues involved in the interaction and their binding energy were predicted by docking guitarrins into the active centers of CmAP and human ALPs.

## 2. Results and Discussion

**Figure 2:**
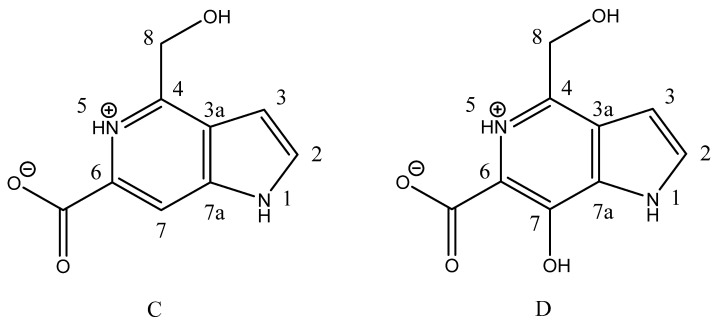
Chemical structures of guitarrin C (C_9_H_8_N_2_O_3_) and D (C_9_H_8_N_2_O_4_).

The screening of 5-azaindoles derived from the marine sponge *G. fimbriata* yielded a pair of effective inhibitors, namely guitarrin C and D (Figure 2), against the highly active alkaline phosphatase CmAP from the marine bacterium *C. amphilecti* KMM 296 [33], which belongs to the protein structural family PhoA, which includes animal and human ALP isoenzymes [36].

**Figure 3:**
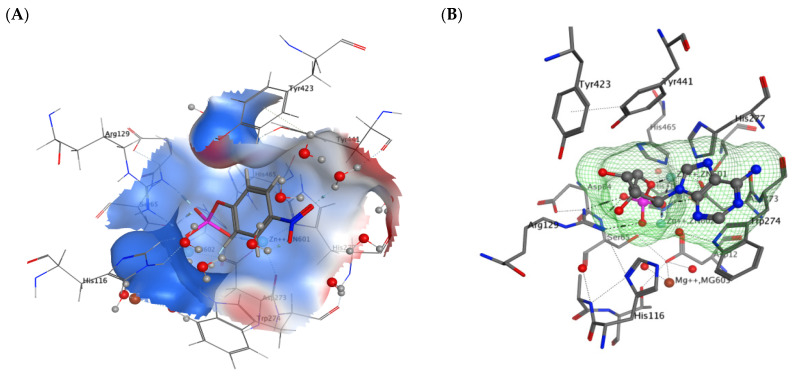
*C. amphilecti* KMM 296 alkaline phosphatase CmAP complexes with the substrates *p*NPP (**A**) and adenosine monophosphate (AMP) (**B**) in the active center, generated by MOE (v. 2020.09). The regions of the active center with an overall positive charge are indicated in blue, while those with an overall negative charge are indicated in red (**A**). The water molecules are depicted as triatomic structures, with the oxygen atom rendered in red and the hydrogen atom in grey. The structures of the substrates and amino acid residues within the active center of the enzyme are illustrated as sticks, with the reactive groups highlighted in red and blue. The amino acid residues that form direct or water-mediated contacts with substrate and metal ions are indicated. The phosphate groups of the substrates are indicated in pink. The catalytic ion Zn^2+^ and the stabilizing ion Mg^2+^ are indicated in blue and brown, respectively. The invisible amino acid residues and metal ions important for catalysis, located behind or within the 3D image of the active site, shown as overlaps and indicated by shadow inscriptions.

**Figure 4:**
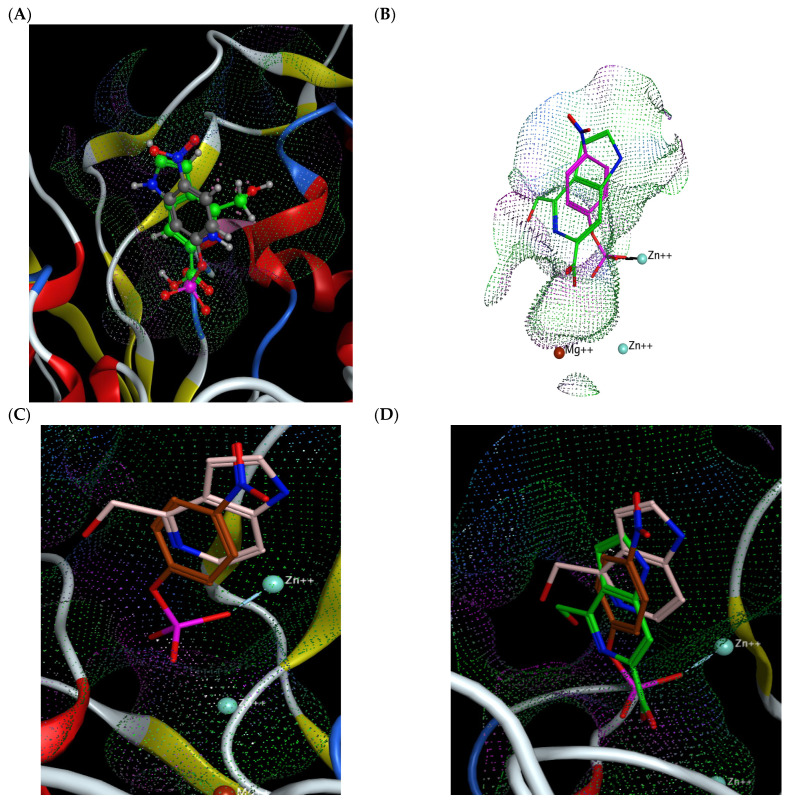
Superimposition of the molecular docking of natural 5-azaindoles into the active site of CmAP in a water molecule environment: (**A**) Guitarrin C (green) and *p*NPP (grey); (**B**) Guitarrin C (green) and *p*NPP (pink); (**C**) Guitarrin B (pink) and *p*NPP (brown); (**D**) Guitarrin C (green), B (pink) and *p*NPP (brown). The reactive end groups are indicated in red and the phosphate group of the substrate *p*NPP is highlighted in pink. Catalytic Zn^2+^ ions are represented by blue spheres, with Mg^2+^ ions represented by brown spheres. The 3D molecular electrostatic potential contour map of the CmAP active site is presented as a network.

In the previous study [33], guitarrins A, B, and E were found to have no inhibitory activity, whereas guitarrins C and D had pronounced inhibitory activity against a highly active alkaline phosphatase, CmAP. This finding indicated that the marine bacterial ALP could serve as a model for the identification of efficacious inhibitors of clinically relevant mammalian ALPs. In addition, the high-quality homology model of the CmAP structure reported earlier [31] was almost identical to the solved structure of the *Vibrio* enzyme (PDB: 3E2D), with a Cα RMSD of 0.43 Å and identical active site metal coordination [27,31]. The superimposition of the molecular docking of the CmAP and VAP (PDB: 6T26) complexes (RMSD = 0.485 Å) (Figures S1–S3) with an aryl chromogenic substrate *p*NPP (Figure 3A), common to all ALPs, and the most effective CmAP inhibitor guitarrin C [33] demonstrated that their molecules compete for the CmAP binding site (Figure 4A).

As a consequence of the similarities in size, three-dimensional structure, orientation, and precise superposition of the carbon atom from the terminal reactive group COOH of the guitarrin C with the substrate POOH group, hydrogen bonds are formed with the catalytic nucleophile Ser 65 via the oxygen atom (Figure 4A).

**Figure 5:**
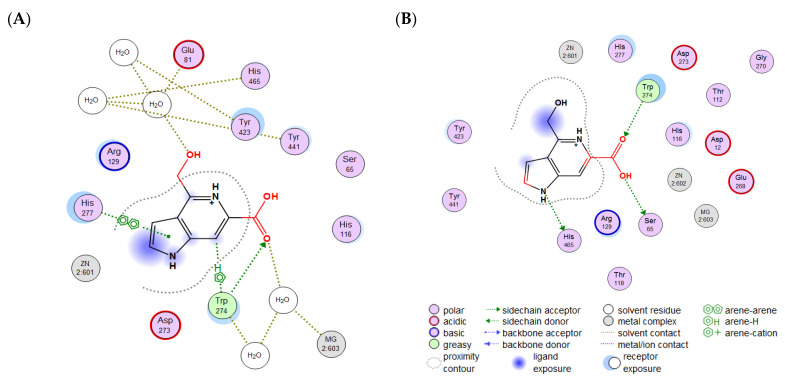

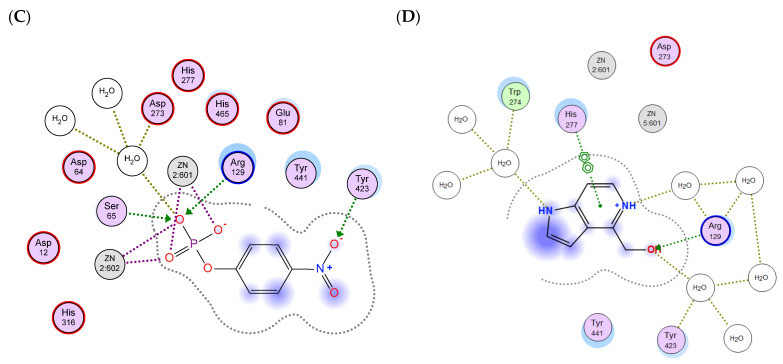
Comparative 2D diagrams of the contacts of guitarrin C (**A**,**B**), *p*NPP (**C**), and guitarrin B (**D**) with amino acid residues of the CmAP active site in a water molecule environment (**A**,**C**,**D**) and without H_2_O in the environment (**B**). Contact symbols are in (**B**) (generated in MOE, v. 2020.09).

However, the introduction of solvent molecules (H_2_O) into the surrounding environment of the enzyme resulted in the guitarrin C molecule becoming integrated into the common hydrogen bond network formed by the atoms of CmAP and H_2_O (Figure 4B). This resulted in a modification of the guitarrin’s interactions within the active site, leading to a transition from precise to approximate superposition with *p*NPP (Figure 4B, Figure 5A,B and Figure S2).

In the presence of H_2_O molecules, the interaction with guitarrin C does not affect the nucleophile Ser65 and the two catalytic ions Zn^2+^ in the active site of CmAP. This is in contrast to the substrate *p*NPP (Figure 4B and Figure 5A,C). While all ALPs interact with *p*NPP by forming contacts with the phosphate ion in the active site via Zn^2+^ to coordinate it with the nucleophilic Ser residue (Figure 5C), it is believed that the Mg^2+^ ion regulates the protonation state of Ser via the water molecules and provides an octahedral geometry to stabilize the most active conformation of the catalytic residues in the ALP dimer [37,38,39]. Furthermore, Mg^2+^ is more likely to be involved in allosteric interactions between subunits of the ALP dimer [37,38].

It can be proposed that in the presence of water and a guitarrin, the catalytic residues of ALP may remain in contact with the substrate *p*NPP, with the limiting step being the release of the reaction product by non-competitive or uncompetitive inhibition [27]. To illustrate, the crystal structure of the marine bacterium *Vibrio splendidus* alkaline phosphatase (VAP) was described in complex with an inhibitory cyclohexylamine, which was employed as a template to model the structures of CmAP (Figure S1). This revealed that the contacts with the inhibitor were formed in the vicinity of the substrate *p*NPP within the active site. However, the kinetic model of catalysis exhibited a non-competitive type of inhibition, as evidenced by the unchanging value of the Michaelis–Menten constant (195 ± 4 μM). This was accompanied by a K_i_ equal to IC_50_ (35.7 mM), with a corresponding decrease in V_max_ [27].

The CmAP complexes exhibited distinctive interactions when the active center was bound to the highly inhibitory guitarrin C and the guitarrin B, which lacks inhibitory activity [33] (Figure 4C,D and Figure 5D). The inhibitor guitarrin C forms contacts with amino acid residues of the active center via a hydrogen network and a key residue, W274, which is involved in Mg^2+^ ion binding and in subunit dimerization and substrate coordination prior to catalysis [27]. The absence of the COOH group on the C6 atom in guitarrin B results in a reduction in the molecular size, which provides a different orientation in relation to the aromatic residues that surround the active center of CmAP. Furthermore, the active center residues that form hydrogen bonds with the heterocycle are also distinct (Figure 4C,D and Figure 5D). It would appear that the formation of hydrogen bonds between guitarrin B and the substrate-binding residue Arg129 is insufficient to inhibit CmAP (Figure 5D). Indeed, the introduction of mutations in the ALP substrate-binding Arg residue did not result in a notable alteration in enzyme activity, as evidenced by the findings of the study [40]. Moreover, the replacement of the residue with Ser provided additional stabilization of the transition state. Similarly, guitarrins A and E, which lack the COOH group and are found in the same marine sponge, also demonstrated no inhibitory activity against CmAP alkaline phosphatase [33].

It is noteworthy that the compound guitarrin D exhibited an order of magnitude lower inhibitory activity towards CmAP than guitarrin C (Table 1). This is likely due to the presence of an additional OH group at carbon atom C7 [33], which impedes the inhibitor molecule from reaching the active site as deeply as guitarrin C (Figure 2, Figure 3 and Figure 4).

**Figure 6:**
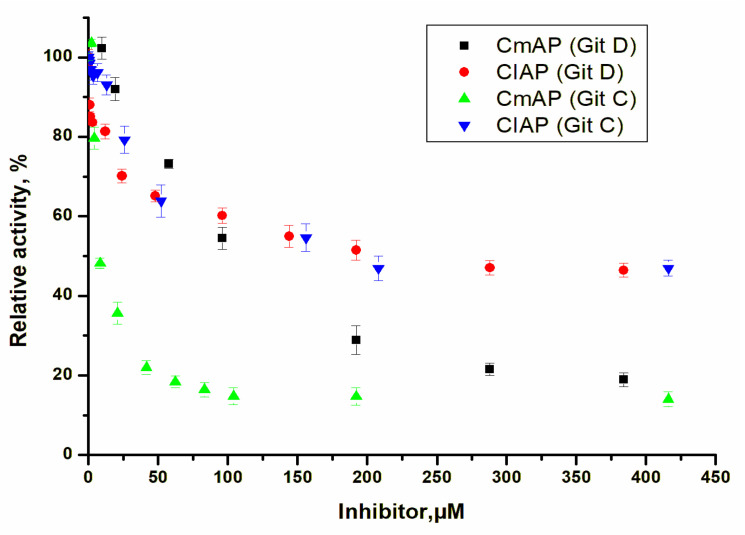
Comparative inhibitory effect of guitarrin C and D on CmAP and CIAP activity. Activity was measured using 2 mM *p*NPP at pH 10.2 and 37 °C in a 100 mM Tris-HCl buffer containing 0.2 M KCL.

The IC_50_ values for guitarrin C and D were 8.5 and 110 µM, respectively, at 50% of the maximum inhibition of the activity of CmAP (85%), resulting in a residual activity of 15% (Table 1, Figure 6). For CIAP, the comparable inhibition constants for guitarrin C and D were determined, and a maximum inhibition of only 57% of the CmAP activity was observed (Table 1, Figure 6).

The marine bacterial ALPs from the Antarctic TAB5 bacterium and *V. splendidus*, for which the enzyme serves as the prototype for the CmAP homology model, were also distinguished from calf intestinal alkaline phosphatases (CAP) and *Escherichia coli* (ECAP) by the inhibitory potency of cyclohexylamine with a non-competitive mode of action [27].

**Figure 7:**
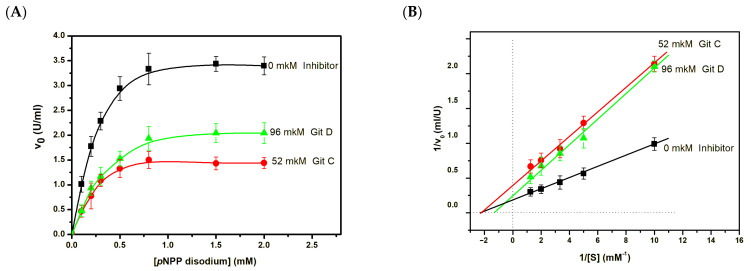
Effect of guitarrin C and D on the V_max_ of *p*NPP (2 mM) catalytic hydrolysis under CmAP (0.003 mg mL^−1^) (**A**). The Linweaver–Burk reciprocal analysis (**B**). The experiments were performed in triplicate.

The determination of the type of inhibition of the CmAP enzyme by the highly specific inhibitor guitarrin C demonstrated that the K_i_ value is less than the IC_50_ value, with a decrease in V_max_ from 5.98 ± 0.03 to 2.62 ± 0.02 U mL^−1^ and an unaltered K_m_ value (0.46 ± 0.2 mM). This is consistent with a non-competitive kinetic model (Table 1, Figure 7). However, guitarrin D exhibited a mixed type of inhibition, with a more than 2-fold decrease in V_max_ from 5.98 ± 0.03 to 3.98 ± 0.02 U mL^−1^ and a 1.6-fold increase in K_m_ from 0.46 ± 0.2 to 0.72 ± 0.1 mM (Table 1, Figure 7).

Furthermore, an analysis of the BRENDA database indicated that mixed-type inhibition is more likely to occur by binding to the active site of the enzyme rather than to the allosteric site of the substrate–enzyme complex, which is a commonly held belief [41]. This type of inhibition is observed in multi-substrate reactions or in reactions involving allosterically regulated enzymes. In their natural environment, marine sponges may regulate ALP activity via 5-azaindoles in order to control cell density and the synthesis of biologically active secondary metabolites, including those produced by the sponges themselves and those produced by bacterial symbionts and pathogens [33,36]. For example, transcriptome analysis of the alkaline phosphatase PhoA in diatoms revealed increased expression of this enzyme coinciding with decreased activity levels of numerous biosynthetic pathways, including iron transport, tetrapyrrole metabolism (particularly chlorophyll), and ATP-dependent proteins [42]. These findings indicate that PhoA is involved in the regulation of the cell cycle and redox processes. It is of interest to note that the CmAP alkaline phosphatase gene is located within the same marine bacterial gene cluster as the electron transport chain proteins, including ATP-dependent NAD^+^-transhydrogenase [36]. This observation indicates that PhoA may perform a similar function in bacteria and eukaryotic cells. Moreover, 5-azaindoles are known to exhibit inhibitory activity against protein kinases involved in cell signaling pathways. This approach has been successfully employed in the development of agents that can be used to switch off aberrant activities of these pathways during oncogenesis [35].

**Figure 8:**
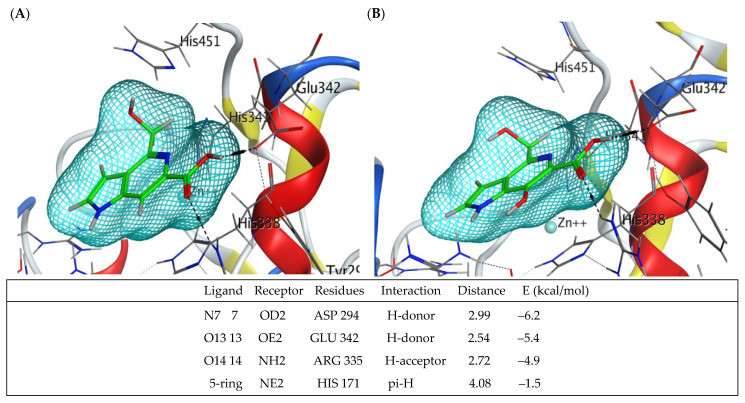
Molecular docking of TNAP alkaline phosphatase with guitarrin C (**A**) and D (**B**). The amino acid residues of the active centre (not all fit in the figure) involved in the interaction with the ligand and coordinating zinc ions are signed and shown as sticks. The remaining enzyme residues are shown as ribbons. The molecules of gittarins C and D are shown as sticks (in green). Interactions between the enzyme and the reactive groups (in blue, red and grey) of the ligands are shown as dashed lines. The 3D molecular electrostatic potential contour map of the ligand binding site is shown as a network (in light blue). The modes of ligand–receptor interactions, distance, and calculated binding energy are presented below (generated by MOE, v. 2020.09).

It can be stated that the presence of a COOH group on the C6 atom and an OH group on the C8 atom is a prerequisite for the inhibitory effect of 5-azoindoles on ALP activity (Figure 2). This assertion is also applicable to mammalian enzymes belonging to the PhoA structural family (Table 1). Nevertheless, guitarrin D revealed that the inhibitory activity against CIAP exhibited a mere 1.2-fold difference compared to that of guitarrin C (Table 1). It seems reasonable to posit that the active site of mammalian ALPs has a larger entrance diameter than that of bacterial CmAP [31] (Figure 3, Figure 8 and Figure S4). However, within 4 Å of the two inhibitory ligands L-phenylalanine (L-Phe) and cyclohexylamine in the alignment of human placental (PLAP) and marine bacterial (VAP) ALPs, respectively, were the conserved metal coordination residues [27]. Therefore, a similar binding mode of the ligands in the presence of bound inorganic phosphate could be expected in VAP and CmAP (Figures S1–S3).

As the crystal structure of calf intestinal ALP has yet to be determined, the binding contacts of guitarrins C and D were analyzed by molecular docking using recently resolved human tissue-nonspecific ALP isoenzyme TNAP [26] and the PLAP-based modelled IAP. The oligomeric interfaces of PLAP and TNAP were found to exhibit significant differences, particularly in the presence of reverse charged or missing residues in PLAP [26]. This indicates that the N-terminal helix performs a supplementary function in TNAP, in addition to that observed in PLAP. These interfacial differences result in the formation of the functional octamer, which exerts a stabilizing effect on the dimer interfaces. This is likely to be of great importance in the context of osteogenesis. Nevertheless, given that PLAP and TNAP have a 55% sequence identity, they display considerable similarity in terms of their overall folding and metal binding/catalytic sites [26].

Following the minimization of energy during the molecular docking of guitarrin D with the TNAP active site, the contacts of the reactive group COO- remain with the same key residues of the active site (Asp 294, Glu 342, Arg 335, and His 171) that are involved in the interaction with guitarrin C (Figure 8 and Figure S5). However, the orientation of the molecule undergoes a slight alteration due to the presence of an additional hydroxyl group at the C7 atom, resulting in an enhancement of the calculated binding energy from −8 to −6 kcal/mol (Figure 8). It is notable that the residues Asp 294 and His 171 have been identified as coordinating with both metal ions and the substrate. Single mutations at these residues have been demonstrated to significantly reduce the relative enzymatic activity of TNAP [26].

It may, therefore, be proposed that further investigation of both guitarrins C and D as inhibitory regulators of ALP isoenzymes in animals and humans may prove beneficial. Moreover, both bacterial and mammalian alkaline phosphatase (ALP) have been observed to demonstrate the highest affinity for their natural purinergic substrates, particularly adenosine monophosphate (AMP) (Figure 3B) [7,43,44]. The substrate preference of bacterial PhoA enzymes remains uncertain [43,44]. However, the physiological role of mammalian ALP is to reduce the expression of pro-inflammatory signaling metabolites through the hydrolysis of purine nucleotides and the production of anti-inflammatory adenosine [7,8,26,45]. As illustrated in Figure 2, Figure 3, Figure 4 and Figure 5, due to their structural similarity, guitarrin C and D can directly compete with AMP for binding sites in the active site of ALP, thereby causing competitive inhibition of the enzymes and the limiting the process of the formation of the substrate–enzyme complex [27]. Nevertheless, the inhibitory activity of the 5-azoindoles, guitarrin C and D, and the kinetic model for human ALP isoenzymes remain to be determined experimentally in order to ascertain their practical efficacy.

## 3. Materials and Methods

### 3.1. Homology Modelling and Molecular Docking

Three-dimensional structures of the guitarrins C and D were constructed and optimized using the molecular modelling software Molecular Operating Environment (MOE), version 2020.09, developed by the Chemical Computing Group Inc. (1010 Sherbooke St. West, Suite #910, Montreal, QC, Canada, H3A 2R7, 2020) [46]. A homology model of alkaline phosphatase CmAP from the marine bacterium *C. amphilecti* KMM 296 was constructed using the high-quality crystal structures (1.4 Å) of *Vibrio* alkaline phosphatase (VAP) (PDB: 3E2D; 6T26), with the co-crystallized inhibitory ligands sulphate and cyclohexylamine, respectively [27,31]. The homology model of the intestinal-type alkaline phosphatase (IAP, Uniprot ID P09923) was obtained from the Swiss-Model server [47,48] using the crystal structure of placental alkaline phosphatase (PLAP, PDB: 3mk1) as a template. Molecular docking was conducted using the Dock module of the MOE program. Docking was performed for the active sites of the alkaline phosphatases CmAP, TNAP (PDB: 7yiv), and IAP using the Score London dG function, with 30 poses initially generated and subsequently refined within 4.5 Å of the placed ligand using the GBVI/WSA dG function [49]. Analysis of the ligand contacts in the complexes was performed using the Ligand Interaction module of the MOE program.

### 3.2. Production of Recombinant Phosphatase CmAP

The *E. coli* Rosetta strain (DE3) was transformed by the plasmid pET40 (b+) containing the gene encoding for the mature alkaline phosphatase CmAP from the marine bacterium *C. amphilecti* KMM 296 [31]. The transformed cells were grown in 25 mL of liquid LB medium containing 50 μg/mL kanamycin at 200 rpm at 37 °C for 16 h. Subsequently, the cells were transferred to fresh LB medium (1 L) containing 50 μg/mL kanamycin and incubated at 37 °C on a shaker at 200 rpm until they reached an optical density at 600 nm of 0.6–0.8. Subsequently, 0.2 mM IPTG was added to induce recombinant protein CmAP expression, and incubation was continued at 18 °C for 18 h at 200 rpm. The cells were precipitated by centrifugation at 4000 rpm for 15 min at 8 °C. They were then suspended in 20 mL of 50 mM Tris-HCl buffer (pH 8.0) and subjected to ultrasonic treatment on a Bandeline ultrasonic disintegrator (Berlin, Germany) at 100% power (22 kHz) and 0–4 °C in 40-s pulses until the suspension was clear. Subsequently, the suspension was subjected to centrifugation at 11,000 rpm for 30 min at 8 °C. The precipitate was discarded, and the CmAP phosphatase activity was determined in the resulting extract. The protein concentration was determined by the Bradford method, with bovine serum albumin (BSA) employed as the reference standard [50].

### 3.3. Isolation and Purification of Recombinant Phosphatase CmAP

For the isolation of CmAP, the resulting supernatant was applied to a 25 × 3.2 cm Ni-IMAC-Sepharose column (Cytiva (GE Healthcare) Life Sciences, Buckinghamshire, UK) that had been equilibrated with 50 mM Tris-HCl, pH 8.0 (buffer A), and washed with five volumes of the same buffer. The recombinant protein was eluted with a linear gradient of 0–0.5 M imidazole in 50 mM Tris-HCl buffer, pH 8.0, and 0.5 M NaCl (6 column volumes) at a rate of 3 mL/min. The fraction containing CmAP was purified on a 10 × 1.4 cm Source 15 Q column (Cytiva (GE Healthcare) Life Sciences, Buckinghamshire, UK) that had been equilibrated with buffer A containing 2 mM MgCl_2_ (buffer B). The protein was then eluted with a linear gradient of 0–0.5 M NaCl in buffer B. Ion exchange chromatography was performed at a rate of 1 mL/min, with the volume of the fractions being 1 mL. The fractions containing CmAP were collected and treated with L-HEP enterokinase at a final concentration of 1 U per 1 mg protein for 18 hs at 25 °C with constant stirring in a vortex. Subsequently, the protein solution was applied to a HisTrap™ high-performance column (Cytiva (GE Healthcare) Life Sciences, Buckinghamshire, UK) that had been pre-equilibrated with buffer A. The recombinant protein was eluted with 10 volumes of buffer B. Fractions containing CmAP were collected and subjected to concentration by ion-exchange chromatography using a Mono-Q HR column (4 × 0.8 cm) (Cytiva (GE Healthcare) Life Sciences, Buckinghamshire, UK). The column was then equilibrated with buffer B and washed with 10 volumes of buffer B, and the target protein was eluted with a linear gradient of 0–0.5 M NaCl in buffer B at a rate of 0.5 mL/min, resulting in 1 mL fractions. The specific activity of CmAP was determined in the fractions obtained.

### 3.4. Alkaline Phosphatase Activity Assay

The enzymatic activity was evaluated through the addition of 5 μL of the protein solution sample to a solution of 495 μL buffer, comprising 0.1 M Tris-HCl (pH 10.0), 0.2 M KCl, and 2 mM *p*NPP. The reaction mixture was incubated at 37 °C for a period of 30 min. To halt the reaction, 2 mL of chilled 0.5 M NaOH was introduced. The quantity of p-nitrophenol (*p*NP) produced was quantified spectrophotometrically at a wavelength of 400 nm, with the control sample, which lacked the enzyme, serving as a reference point. The specific activity was calculated using the following formula: (2.5 × OD400)/(18.6 × 0.005 × t × C), where OD400 is the optical density at a wavelength of 400 nm, 2.5 is the volume of the reaction mixture in mL, 18.6 is the extinction coefficient of *p*NP (M^−1^/cm^−1^), t is the incubation time at 37 °C in minutes, 0.005 is the sample volume in mL, and C is the protein concentration in the sample (mg/mL). The quantity of enzyme required for the generation of 1 µM *p*NP within a one-minute period was established as the unit of activity.

### 3.5. Inhibitory Activity Assay

The effect of the natural inhibitors guitarrins C and D [33] on the activity of the alkaline phosphatases CmAP from *C. amphilecti* KMM 296 and calf intestinal alkaline phosphatase (CIAP, Invitrogen™ 18009019, Thermo Fisher Scientific, Waltham, MA, USA) was investigated in a buffer containing 0.1 M tris-HCl (pH 10.0) with 0.2 KCl. For this purpose, 5 μL of enzyme, 20 μL of inhibitor of different concentrations (0–10.4 µM), and 470 μL of the indicated buffer were added to an incubation mixture totaling 500 μL and incubated for 60 min, and then the enzymatic reaction was initiated by adding 5 μL of *p*NPP substrate. The activity of ALPs was determined using the standard method described above. CmAP (3.22 mg/mL, 2929 U/mg) and CIAP (0.99 mg/mL, 647 U/mg) were dissolved in 50 mM tris-HCl (pH 8.0) buffer with 2 mM MgCl_2_. Results were presented as the percentage of inhibition relative to control activity. The IC_50_ for each compound tested was defined as the concentration of compound that inhibited the enzyme activity by 50%. IC_50_ values are given as the mean of three experiments and standard deviation.

### 3.6. Determination of Inhibition Type and Constant (K_i_)

The kinetic parameters (K_m_ and V_max_) were determined for alkaline phosphatases CmAP and CIAP. The mean K_m_ values and standard deviations were presented. In order to ascertain the nature of the inhibition, the K_m_ was determined in the presence of guitarrin C (52.0 µM) and guitarrin D (96.0 µM) by preincubating CmAP with the inhibitor for 60 min at room temperature (23 °C). The inhibition constant, K_i_, was determined using the Cheng–Prusoff formula [51]. The binding activity of the inhibitor to the reactive substrate (Ki) can be calculated using the following formula: Ki = IC_50_/(1 + [S]/Km), where IC_50_ is the functional activity of the inhibitor, [S] is the concentration of the reactive substrate, and KM is the Michaelis constant. The results were presented in the Origin 7.0 software.

## 4. Conclusions

The kinetic parameters and chemical nature of the inhibitory effect of natural 5-azaindoles, guitarrin C and D derived from the marine sponge, on the alkaline phosphatase CmAP from the marine bacterium were determined. Guitarrin C was identified as a highly selective non-competitive inhibitor of the marine enzyme due to the presence of a carboxyl group at C6 and a hydroxyl group at C8, with an IC_50_ of 8.5 ± 0.08 µM and a K_i_ of 1.58 ± 0.04 µM. It was hypothesized that this compound acts as an allosteric regulator of enzymes present in both resident and pathogenic sponge microflora. The non-competitive inhibitor would form contacts with amino acid residues of the active center via a hydrogen network and a key residue, W274, which is involved in Mg^2+^ ion binding and in subunit dimerization and substrate coordination prior to catalysis. However, the less active inhibitor, guitarrin D, which features an additional hydroxyl group at C7, demonstrated a mixed type of inhibition, which may also have regulatory implications.

It was proposed that both 5-azaindoles may interact with the active site of human ALPs, thereby modulating their function by forming hydrogen bonds with the nitrogen and oxygen atoms of the guitarrin C and D heterocycles. This hypothesis is based on the relatively low calculated binding energy between the key human ALP residues Asp 294, Glu 342, Arg 335, and His 171, which are situated in proximity to and coordinating with the catalytic metal ions, as well as the inhibitory activity of both guitarrins towards the animal ALP isoenzyme. The IC_50_ value for the bovine ALP isoenzyme in the presence of guitarrin C was found to be 195 ± 2.2 µM, with a K_i_ value of 38 ± 0.1 µM. Similarly, the IC_50_ value for guitarrin D was determined to be 230 ± 1.6 µM, with a K_i_ value of 45 ± 0.2 µM.

## Figures and Tables

**Table 1:** Kinetic and inhibition values for alkaline phosphatases CIAP and CmAP.

Value	CIAP	CmAP
IC_50_ (Guit C) *	195 ± 2.2 µM	8.5 ± 0.08 µM
IC_50_ (Guit D) **	230 ± 1.6 µM	110 ± 0.8 µM
K_i_ (Guit C) *	38 ± 0.1 µM	1.58 ± 0.04 µM
K_i_ (Guit D) *	45 ± 0.2 µM	20.56 ± 0.06 µM
V_max_	0.35 ± 0.012 U mL^−1^	5.98 ± 0.03 U mL^−1^
V_1/2_	0.175 ± 0.007 U mL^−1^	2.99 ± 0.01 U mL^−1^
K_m_	0.49 ± 0.015 mM	0.46 ± 0.2 mM

*, guitarrin C; **, guitarrin D.

## Data Availability

Data are contained within the article and Supplementary Materials.

## Supplementary Materials

The following supporting information can be downloaded at: https://www.mdpi.com/article/10.3390/molecules29235701/s1, Figure S1: Molecular docking of *Vibrio* alkaline phosphatase VAP (PDB: 6T26) and the inhibitor cyclohexylamine using MOE v. 2020.09. The key residue interactions with the ligand are consistent with those of experimental VAP model described in [27]; Figure S2: Ligand interactions in the superimposed complexes of CmAP with *p*NPP substrate and guitarrin C inhibitor (MOE v. 2020.09). Ligand interaction report is below; Figure S3: Active sites binding of *p*NPP (dark yellow sticks) and the inhibitory ligands guitarrin C (green sticks), and cyclohexylamine (light yellow sticks) to the bacterial alkaline phosphatases CmAP and VAP (PDB: 6T26.A, [27]) aligned (shown as ribbons). The active site zinc ions are shown as blue spheres. The superposition report for CmAP (1: ap) and VAP (3: 6T26.A) is below; Figure S4: Electrostatic surface potentials of the TNAP entrance to the active site bound with guitarrin C. Positively charged amino acid residues are indicated by blue color. Negatively charged amino acid residues are indicated by red color (generated in MOE, v. 2020.09); Figure S5: 2-D diagram of the contacts of guitarrin C and human alkaline phosphatase TNAP. The mode of ligand-receptor interactions, the distance and the calculated binding energy are presented in the table below (generated in MOE, v. 2020.09).
