# Supplementary material for: In Silico Prediction of Alkaline Phosphatase Interaction with the Natural Inhibitory 5-Azaindoles Guitarrin C and D
Source: Molecules. 2024 Dec 3;29(23):5701. doi: 10.3390/molecules29235701 (PMC11643677; doi:10.3390/molecules29235701)
Supplement: Supplementary file 1 [file molecules-29-05701-s001.zip › molecules-3312536-supplementary.pdf]

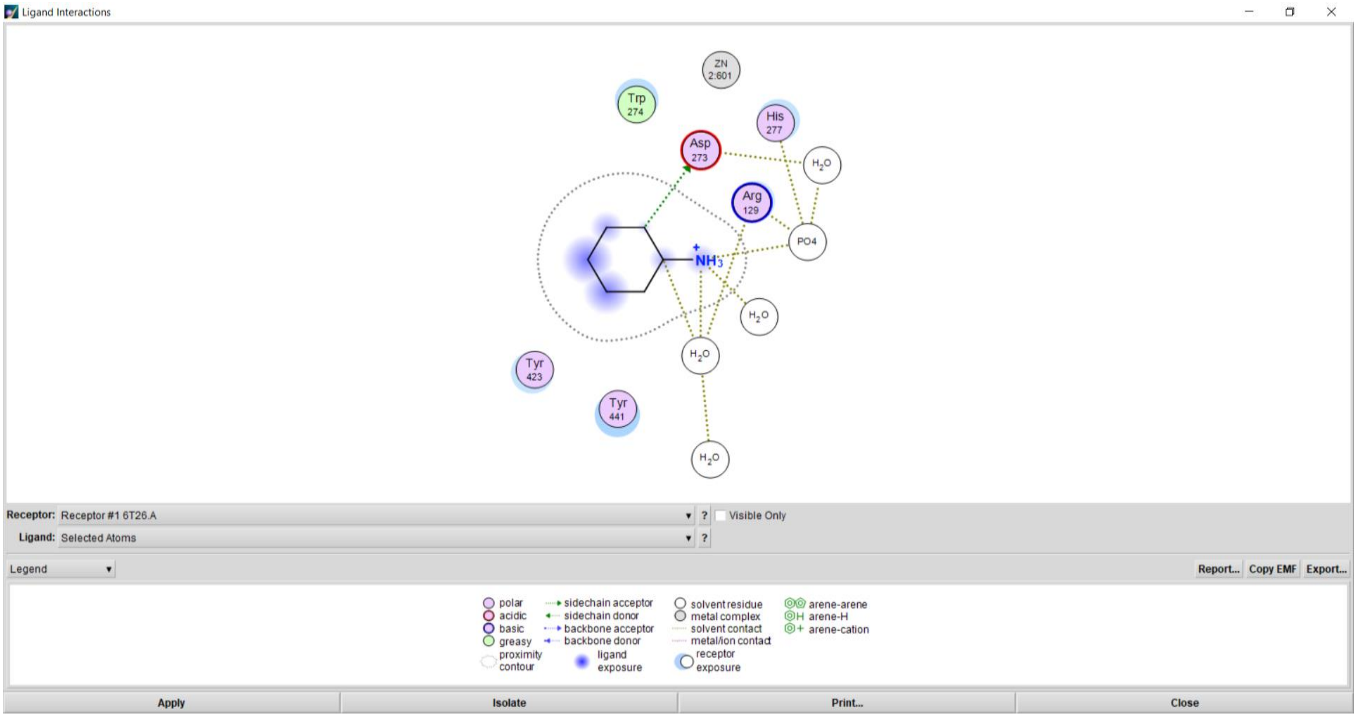

Figure S1. Molecular docking of *Vibrio* alkaline phosphatase VAP (PDB:6T26) and the inhibitor cyclohexylamine using MOE v.2020.09. The key residue interactions with the ligand are consistent with those of experimental VAP model described in [27].

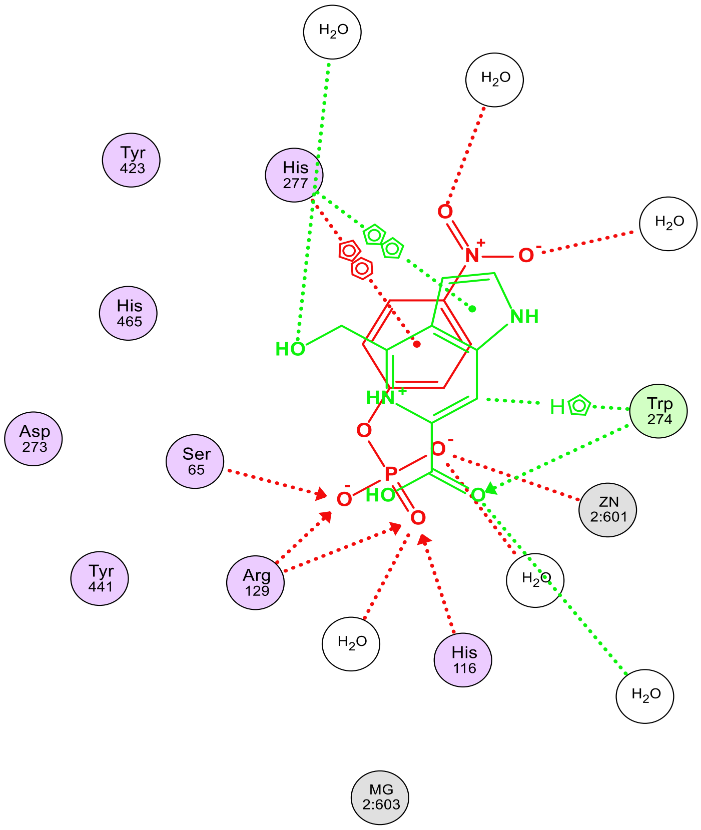

Figure S2. The Ligand interactions in the superimposed complexes of CmAP with *p*NPP substrate and Guittarin C inhibitor (MOE v.2020.09). Ligand interaction report is below.

Ligand Interactions Report

CmAP/pNPP complex

Ligand		Receptor				Interaction	Distance	E (kcal/mol)
O2	3	CE1	HIS	116		H-acceptor	3.34	-0.6
O2	3	NH2	ARG	129		H-acceptor	2.53	-8.5
O2	3	O	HOH	629	(A)	H-acceptor	2.91	-2.1
O3	4	O	HOH	624	(A)	H-acceptor	2.89	-3.4
O4	5	OG	SER	65		H-acceptor	2.70	-4.3
O4	5	NH1	ARG	129		H-acceptor	2.56	-8.8
O5	13	O	HOH	1009	(A)	H-acceptor	2.89	-1.4
O6	14	O	HOH	1045	(A)	H-acceptor	2.89	-0.8
O3	4	ZN	ZN	601	(A)	Metal	1.82	-4.8
O2	3	NH1	ARG	129		Ionic	3.68	-1.3
O2	3	NH2	ARG	129		Ionic	2.53	-8.5
O3	4	ZN	ZN	601	(A)	Ionic	1.82	-20.0
O4	5	NH1	ARG	129		Ionic	2.56	-8.2
O4	5	NH2	ARG	129		Ionic	3.22	-3.2
ZN	601	O	ASP	273		Metal	2.06	-3.4
ZN	601	OD2	ASP	273		Metal	1.92	-4.1
ZN	601	ND1	HIS	277		Metal	2.10	-2.0
O	629	MG	MG	603	(A)	Metal	2.30	-1.7
ZN	601	OD2	ASP	273		Ionic	1.92	-17.9
6-ring		5-ring	HIS	277		pi-pi	3.91	-0.0

(**CmAP/Guittarin C**)

Ligand		Receptor				Interaction	Distance	E (kcal/mol)
O2	4	O	HOH	679	(A)	H-donor	2.66	-1.9
O	13	NE1	TRP	274		H-acceptor	3.07	-1.8
O	13	O	HOH	648	(A)	H-acceptor	3.28	-0.6
ZN	601	O	ASP	273		Metal	2.06	-3.4
ZN	601	OD2	ASP	273		Metal	1.92	-4.1
ZN	601	ND1	HIS	277		Metal	2.10	-2.0
O	648	MG	MG	603	(A)	Metal	2.14	-2.1
ZN	601	OD2	ASP	273		Ionic	1.92	-17.9
C	7	5-ring	TRP	274		H-pi	4.22	-0.7

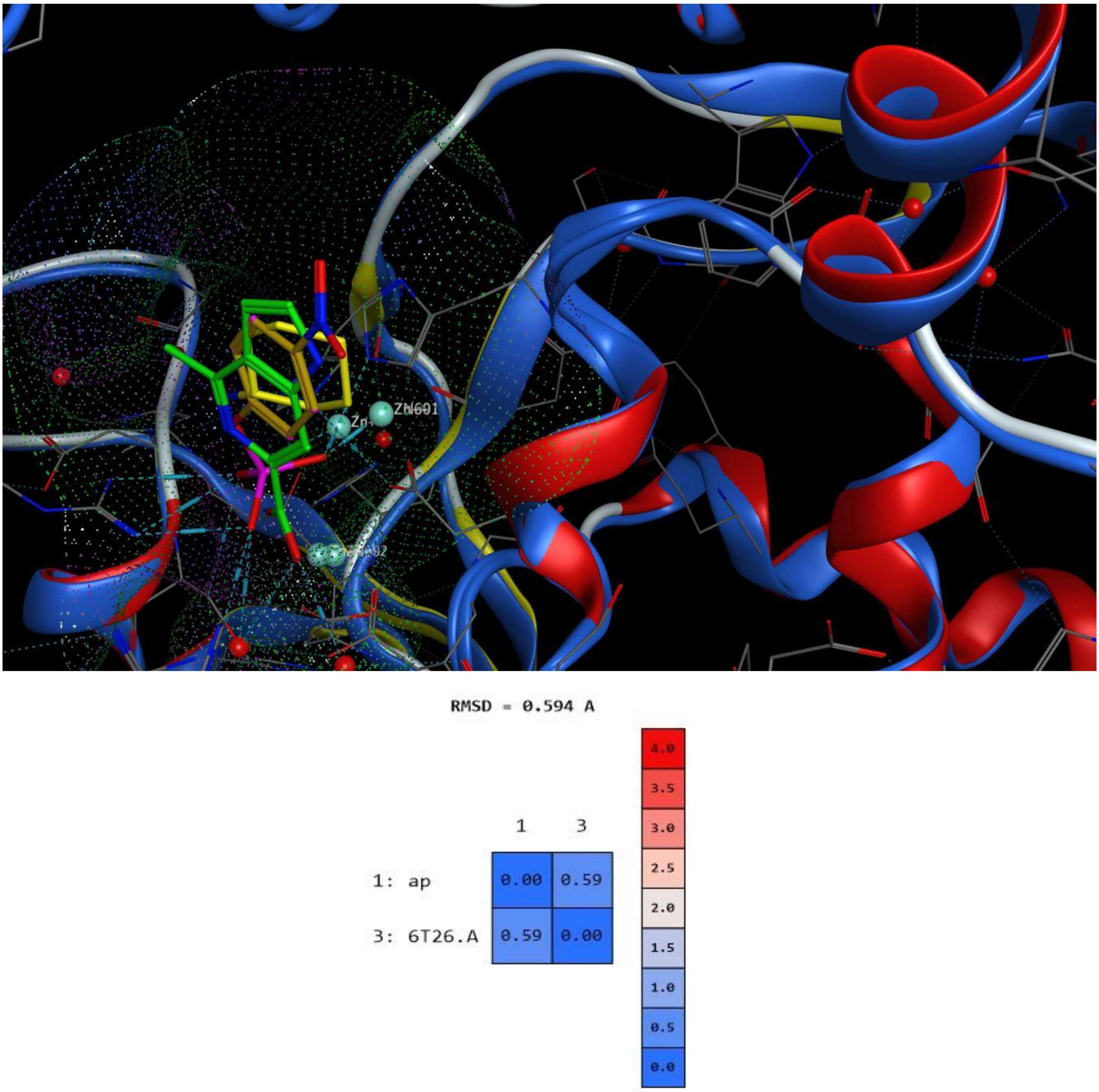

Figure S3. Active sites binding of *p*NPP (dark yellow sticks) and the inhibitory ligands guitarrin C (green sticks), and cyclohexylamine (light yellow sticks) to the bacterial alkaline phosphatases CmAP and VAP (PDB: 6T26.A, [27]) aligned (shown as ribbons). The active site zinc ions are shown as blue spheres. The superposition report for CmAP (1: ap) and VAP (3: 6T26.A) is below.

Pairwise RMSD Matrix:

Chains 1: ap – CmAP 3: 6T26.A - VAP

1: CmAP HYDROLASE

vs.

3: 6T26.A HYDROLASE

RMSD = 0.594 A

ALA 1 - ALA 1 : 0.432

GLU 2 - GLU 2 : 1.382

ILE 3 - ILE 3 : 0.947

LYS 4 - LYS 4 : 0.441

ASN 5 - ASN 5 : 0.315

VAL 6 - VAL 6 : 0.270

ILE 7 - ILE 7 : 0.207

LEU 8 - LEU 8 : 0.093

MET 9 - MET 9 : 0.336

ILE 10 - ILE 10 : 0.142

GLY 11 - GLY 11 : 0.281

ASP 12 - ASP 12 : 0.354

GLY 13 - GLY 13 : 0.216

MET 14 - MET 14 : 0.363

GLY 15 - GLY 15 : 0.531

PRO 16 - PRO 16 : 0.361

GLN 17 - GLN 17 : 0.605

GLN 18 - GLN 18 : 0.415

VAL 19 - VAL 19 : 0.367

GLY 20 - GLY 20 : 0.408

LEU 21 - MET 21 : 0.308

LEU 22 - LEU 22 : 0.205

GLU 23 - GLU 23 : 0.076

THR 24 - THR 24 : 0.449

TYR 25 - TYR 25 : 0.250

ALA 26 - ALA 26 : 0.331

ASN 27 - ASN 27 : 0.298

GLN 28 - ARG 28 : 0.295

ALA 29 - ALA 29 : 0.248

PRO 30 - PRO 30 : 0.425

ASN 31 - ASP 31 : 1.000

SER 32 - SER 32 : 0.527

ILE 33 - ILE 33 : 0.325

TYR 34 - TYR 34 : 0.223

LYS 35 - GLN 35 : 0.422

GLY 36 - GLY 36 : 0.878

ASN 37 - ARG 37 : 0.498

LYS 38 - SER 38 : 0.170

THR 39 - THR 39 : 0.100

ALA 40 - ALA 40 : 0.277

ILE 41 - LEU 41 : 0.225

TYR 42 - TYR 42 : 0.263

GLN 43 - GLN 43 : 0.834

LEU 44 - LEU 44 : 0.580

ALA 45 - ALA 45 : 0.507

GLN 46 - LYS 46 : 0.925

GLU 47 - GLU 47 : 0.366

GLY 48 - GLY 48 : 0.489

VAL 49 - VAL 49 : 0.382

ILE 50 - VAL 50 : 0.447

GLY 51 - GLY 51 : 0.915

SER 52 - ALA 52 : 0.359

SER 53 - SER 53 : 0.220

LEU 54 - LEU 54 : 0.239

THR 55 - THR 55 : 0.203

HIS 56 - HIS 56 : 0.226

PRO 57 - PRO 57 : 0.346

GLU 58 - GLU 58 : 0.285

ASP 59 - ASP 59 : 0.530

ALA 60 - ALA 60 : 0.360

ILE 61 - VAL 61 : 0.687

VAL 62 - VAL 62 : 0.489

VAL 63 - VAL 63 : 0.472

ASP 64 - ASP 64 : 0.269

SER 65 - SER 65 : 0.395

ALA 66 - ALA 66 : 0.285

CYS 67 - CYS 67 : 0.243

SER 68 - SER 68 : 0.297

ALA 69 - ALA 69 : 0.248

THR 70 - THR 70 : 0.379

MET 71 - GLN 71 : 0.220

LEU 72 - LEU 72 : 0.203

ALA 73 - SER 73 : 0.162

THR 74 - THR 74 : 0.715

GLY 75 - GLY 75 : 0.582

ILE 76 - ILE 76 : 0.180

TYR 77 - PHE 77 : 0.270

SER 78 - THR 78 : 0.231

SER 79 - GLY 79 : 0.370

SER 80 - GLY 80 : 0.274

GLU 81 - GLU 81 : 0.265

VAL 82 - VAL 82 : 0.406

ILE 83 - ILE 83 : 0.300

GLY 84 - GLY 84 : 0.103

ILE 85 - ILE 85 : 0.126

ASP 86 - ASP 86 : 0.056

SER 87 - SER 87 : 0.163

GLN 88 - GLU 88 : 0.231

GLY 89 - GLY 89 : 0.928

ASN 90 - ASN 90 : 0.550

HIS 91 - ARG 91 : 0.433

VAL 92 - VAL 92 : 0.464

GLU 93 - GLU 93 : 0.419

THR 94 - THR 94 : 0.189

VAL 95 - VAL 95 : 0.338

LEU 96 - LEU 96 : 0.429

GLU 97 - GLU 97 : 0.325

LYS 98 - LEU 98 : 0.359

ALA 99 - ALA 99 : 0.406

LYS 100 - LYS 100 : 0.529

LYS 101 - ARG 101 : 0.892

ALA 102 - VAL 102 : 0.750

GLY 103 - GLY 103 : 0.650

LYS 104 - LYS 104 : 0.493

ALA 105 - ALA 105 : 0.395

THR 106 - THR 106 : 0.452

GLY 107 - GLY 107 : 0.405

LEU 108 - LEU 108 : 0.437

VAL 109 - VAL 109 : 0.494

SER 110 - SER 110 : 0.438

ASP 111 - ASP 111 : 0.304

THR 112 - THR 112 : 0.601

ARG 113 - ARG 113 : 0.470

LEU 114 - LEU 114 : 0.338

THR 115 - THR 115 : 0.300

HIS 116 - HIS 116 : 0.382

ALA 117 - ALA 117 : 0.352

THR 118 - THR 118 : 0.180

PRO 119 - PRO 119 : 0.249

ALA 120 - ALA 120 : 0.314

SER 121 - ALA 121 : 0.255

PHE 122 - PHE 122 : 0.357

ALA 123 - ALA 123 : 0.290

ALA 124 - ALA 124 : 0.139

HIS 125 - HIS 125 : 0.301

GLN 126 - GLN 126 : 0.203

PRO 127 - PRO 127 : 0.319

HIS 128 - HIS 128 : 0.406

ARG 129 - ARG 129 : 0.441

SER 130 - SER 130 : 0.341

LEU 131 - LEU 131 : 0.593

GLU 132 - GLU 132 : 0.302

ASN 133 - ASN 133 : 0.475

GLN 134 - ALA 134 : 0.433

ILE 135 - ILE 135 : 0.298

ALA 136 - ALA 136 : 0.375

SER 137 - GLU 137 : 0.814

ASP 138 - ASP 138 : 0.789

MET 139 - MET 139 : 0.256

LEU 140 - LEU 140 : 0.391

ALA 141 - MET 141 : 0.517

THR 142 - THR 142 : 0.436

GLY 143 - GLY 143 : 0.512

ALA 144 - PRO 144 : 0.219

ASP 145 - ASP 145 : 0.352

VAL 146 - VAL 146 : 0.381

MET 147 - MET 147 : 0.339

LEU 148 - LEU 148 : 0.660

SER 149 - SER 149 : 0.619

GLY 150 - GLY 150 : 0.501

GLY 151 - GLY 151 : 0.583

LEU 152 - LEU 152 : 0.360

ARG 153 - ARG 153 : 0.479

HIS 154 - HIS 154 : 0.771

TRP 155 - PHE 155 : 0.621

ILE 156 - VAL 156 : 0.512

PRO 157 - PRO 157 : 0.629

LYS 158 - GLN 158 : 0.435

SER 159 - SER 159 : 0.818

THR 160 - VAL 160 : 0.691

ASN 161 - SER 161 : 0.825

ASP 162 - GLU 162 : 0.942

LYS 163 - PRO 163 : 1.016

GLY 164 - GLY 164 : 0.998

GLU 165 - GLU 165 : 1.044

THR 166 - SER 166 : 0.810

TYR 167 - ALA 167 : 0.873

LYS 168 - GLY 168 : 1.168

GLN 169 - SER 169 : 0.804

LEU 170 - VAL 170 : 0.436

GLU 171 - GLU 171 : 0.765

LYS 172 - THR 172 : 0.720

LEU 173 - LEU 173 : 0.487

THR 174 - MET 174 : 0.564

GLN 175 - GLN 175 : 0.598

GLY 176 - GLY 176 : 0.603

ASP 177 - ALA 177 : 0.296

VAL 178 - TRP 178 : 1.426

TYR 179 - SER 179 : 1.187

LEU 180 - PRO 180 : 1.503

LYS 181 - THR 181 : 0.823

SER 182 - SER 182 : 0.505

LYS 183 - LYS 183 : 0.405

ARG 184 - ARG 184 : 0.217

LYS 185 - LYS 185 : 0.641

ASP 186 - ASP 186 : 0.743

ASP 187 - GLU 187 : 0.649

ARG 188 - ARG 188 : 0.581

ASN 189 - ASN 189 : 0.480

LEU 190 - LEU 190 : 0.242

LEU 191 - LEU 191 : 0.337

THR 192 - GLN 192 : 0.228

GLU 193 - GLU 193 : 0.244

ALA 194 - ALA 194 : 0.204

GLU 195 - ALA 195 : 0.348

LYS 196 - ASP 196 : 0.398

ASP 197 - GLN 197 : 0.344

GLY 198 - GLY 198 : 0.401

TYR 199 - TYR 199 : 0.408

GLN 200 - GLY 200 : 0.394

LEU 201 - LEU 201 : 0.730

ALA 202 - ALA 202 : 0.709

PHE 203 - PHE 203 : 0.454

ASN 204 - THR 204 : 1.052

ARG 205 - ARG 205 : 0.367

ASN 206 - ASP 206 : 0.496

MET 207 - GLN 207 : 0.348

LEU 208 - MET 208 : 0.223

ASP 209 - ALA 209 : 0.421

ASP 210 - ALA 210 : 0.491

ALA 211 - LEU 211 : 0.725

LYS 212 - ASN 212 : 1.214

GLY 213 - GLY 213 : 0.783

ASP 214 - THR 214 : 0.641

LYS 215 - LYS 215 : 0.491

LEU 216 - VAL 216 : 0.529

LEU 217 - LEU 217 : 0.067

GLY 218 - GLY 218 : 0.078

LEU 219 - LEU 219 : 0.116

PHE 220 - PHE 220 : 0.247

ALA 221 - ALA 221 : 0.294

TYR 222 - ASN 222 : 0.187

SER 223 - SER 223 : 0.256

GLY 224 - GLY 224 : 0.344

MET 225 - MET 225 : 0.163

ASP 226 - ALA 226 : 0.174

ASP 227 - ASP 227 : 0.259

GLY 228 - GLY 228 : 0.237

ILE 229 - ILE 229 : 0.511

ALA 230 - SER 230 : 0.756

TYR 231 - PHE 231 : 0.354

SER 232 - ARG 232 : 0.333

ASN 233 - ASP 233 : 0.302

LYS 234 - SER 234 : 0.517

LYS 235 - HIS 235 : 0.609

LYS 236 - ASP 236 : 4.101 !

SER 237 - ASP 237 : 3.140 !

GLY 238 - PRO 238 : 3.334 !

GLU 239 - GLN 239 : 2.907 !

ARG 240 - ARG 240 : 1.050

THR 241 - GLN 241 : 0.732

GLN 242 - GLN 242 : 0.252

PRO 243 - PRO 243 : 0.538

SER 244 - THR 244 : 0.599

LEU 245 - LEU 245 : 0.660

LYS 246 - HIS 246 : 0.460

GLU 247 - GLU 247 : 0.380

MET 248 - MET 248 : 0.322

THR 249 - THR 249 : 0.348

GLN 250 - GLN 250 : 0.337

LYS 251 - LYS 251 : 0.171

ALA 252 - ALA 252 : 0.252

LEU 253 - LEU 253 : 0.248

ASN 254 - SER 254 : 0.411

ILE 255 - MET 255 : 0.265

LEU 256 - LEU 256 : 0.051

SER 257 - GLU 257 : 0.312

LYS 258 - GLN 258 : 0.215

ASP 259 - ASP 259 : 0.888

GLU 260 - ASP 260 : 0.966

ASP 261 - ASP 261 : 1.031

GLY 262 - GLY 262 : 0.425

PHE 263 - PHE 263 : 0.344

PHE 264 - PHE 264 : 0.398

LEU 265 - LEU 265 : 0.318

MET 266 - MET 266 : 0.068

VAL 267 - VAL 267 : 0.409

GLU 268 - GLU 268 : 0.548

GLY 269 - GLY 269 : 0.335

GLY 270 - GLY 270 : 0.484

GLN 271 - GLN 271 : 0.145

ILE 272 - ILE 272 : 0.407

ASP 273 - ASP 273 : 0.519

TRP 274 - TRP 274 : 0.313

ALA 275 - ALA 275 : 0.308

GLY 276 - ALA 276 : 0.594

HIS 277 - HIS 277 : 0.859

SER 278 - SER 278 : 0.515

ASN 279 - ASN 279 : 0.410

ASP 280 - ASP 280 : 0.478

ALA 281 - ALA 281 : 0.339

GLY 282 - GLY 282 : 0.617

THR 283 - THR 283 : 0.368

MET 284 - MET 284 : 0.206

LEU 285 - LEU 285 : 0.232

HIS 286 - ASN 286 : 0.274

GLU 287 - GLU 287 : 0.169

LEU 288 - LEU 288 : 0.017

LEU 289 - ILE 289 : 0.352

LYS 290 - LYS 290 : 0.516

PHE 291 - PHE 291 : 0.142

ASP 292 - ASP 292 : 0.176

GLU 293 - GLU 293 : 0.332

ALA 294 - ALA 294 : 0.091

ILE 295 - VAL 295 : 0.201

GLN 296 - GLN 296 : 0.196

THR 297 - GLY 297 : 0.150

VAL 298 - VAL 298 : 0.139

TYR 299 - PHE 299 : 0.269

GLU 300 - ASP 300 : 0.199

TRP 301 - TRP 301 : 0.599

ALA 302 - ALA 302 : 0.847

LYS 303 - ARG 303 : 0.901

ASP 304 - ASP 304 : 0.571

ARG 305 - ARG 305 : 0.463

GLU 306 - ASP 306 : 0.453

ASP 307 - ASP 307 : 0.312

THR 308 - THR 308 : 0.356

ILE 309 - ILE 309 : 0.242

VAL 310 - ILE 310 : 0.188

ILE 311 - LEU 311 : 0.377

VAL 312 - VAL 312 : 0.375

THR 313 - THR 313 : 0.405

ALA 314 - ALA 314 : 0.455

ASP 315 - ASP 315 : 0.449

HIS 316 - HIS 316 : 0.615

GLU 317 - GLU 317 : 0.214

THR 318 - THR 318 : 0.132

GLY 319 - GLY 319 : 0.363

SER 320 - ALA 320 : 0.656

PHE 321 - PHE 321 : 0.560

GLY 322 - GLY 322 : 0.342

PHE 323 - PHE 323 : 0.457

SER 324 - SER 324 : 0.397

TYR 325 - TYR 325 : 0.596

SER 326 - SER 326 : 0.401

SER 327 - SER 327 : 0.271

ASN 328 - ALA 328 : 0.685

ASP 329 - ASN 329 : 0.896

LEU 330 - LEU 330 : 0.501

PRO 331 - PRO 331 : 0.585

LYS 332 - ALA 332 : 0.839

PRO 333 - ALA 333 : 0.335

GLN 334 - GLN 334 : 0.576

LYS 335 - LYS 335 : 0.789

ARG 336 - LYS 336 : 0.494

SER 337 - SER 337 : 0.755

GLY 338 - GLY 338 : 0.446

GLU 339 - PRO 339 : 0.520

ALA 340 - ALA 340 : 0.472

PHE 341 - PHE 341 : 0.296

ALA 342 - ALA 342 : 0.378

ASP 343 - ASP 343 : 0.289

ARG 344 - GLN 344 : 0.681

ASP 345 - ASP 345 : 0.867

TYR 346 - TYR 346 : 0.343

ALA 347 - ALA 347 : 0.249

PRO 348 - PRO 348 : 0.264

ASN 349 - ASN 349 : 0.287

PHE 350 - PHE 350 : 0.549

ASN 351 - ASN 351 : 0.445

PHE 352 - PHE 352 : 0.385

GLY 353 - GLY 353 : 0.566

ALA 354 - ASP 354 : 0.294

PHE 355 - PHE 355 : 0.412

ASP 356 - SER 356 : 0.356

ILE 357 - ILE 357 : 0.193

LEU 358 - LEU 358 : 0.083

ASP 359 - ASP 359 : 0.123

GLY 360 - SER 360 : 0.130

LEU 361 - LEU 361 : 0.267

TYR 362 - TYR 362 : 0.230

ASN 363 - GLU 363 : 0.247

GLN 364 - GLN 364 : 0.551

LYS 365 - LYS 365 : 0.469

GLN 366 - GLN 366 : 0.439

SER 367 - THR 367 : 0.246

TYR 368 - TYR 368 : 0.636

TYR 369 - TYR 369 : 0.455

GLY 370 - GLU 370 : 0.765

MET 371 - LEU 371 : 0.633

ILE 372 - LEU 372 : 0.660

SER 373 - SER 373 : 0.571

GLU 374 - ASP 374 : 0.816

PHE 375 - PHE 375 : 0.731

GLN 376 - GLU 376 : 0.693

LYS 377 - ALA 377 : 1.045

LEU 378 - LEU 378 : 1.256

ASP 379 - PRO 379 : 1.118

LYS 380 - GLN 380 : 1.151

SER 381 - GLY 381 : 1.103

LEU 382 - GLU 382 : 0.426

GLN 383 - ARG 383 : 0.770

THR 384 - THR 384 : 0.692

PRO 385 - PRO 385 : 0.211

GLU 386 - ALA 386 : 0.349

LYS 387 - ARG 387 : 0.468

LEU 388 - LEU 388 : 0.028

ALA 389 - MET 389 : 0.155

GLU 390 - ALA 390 : 0.488

ILE 391 - ALA 391 : 0.314

VAL 392 - VAL 392 : 0.294

ASN 393 - ASN 393 : 0.554

LYS 394 - GLY 394 : 0.723

ASN 395 - ASN 395 : 0.296

SER 396 - SER 396 : 0.525

GLU 397 - ASP 397 : 0.471

PHE 398 - PHE 398 : 0.399

PRO 399 - GLN 399 : 0.629

ILE 400 - ILE 400 : 0.613

THR 401 - THR 401 : 0.277

ALA 402 - GLU 402 : 0.441

GLU 403 - ALA 403 : 0.514

GLN 404 - GLN 404 : 0.497

ALA 405 - ALA 405 : 0.373

LYS 406 - ALA 406 : 0.642

ASN 407 - GLU 407 : 0.380

VAL 408 - VAL 408 : 0.155

LEU 409 - LEU 409 : 0.374

ALA 410 - ALA 410 : 0.326

SER 411 - ASN 411 : 0.248

LYS 412 - LYS 412 : 0.270

PRO 413 - PRO 413 : 0.281

ASN 414 - ASN 414 : 0.309

PRO 415 - PRO 415 : 0.505

TYR 416 - TYR 416 : 0.429

ARG 417 - HIS 417 : 0.152

LEU 418 - VAL 418 : 0.130

ALA 419 - ASP 419 : 0.420

GLN 420 - GLY 420 : 0.257

HIS 421 - HIS 421 : 0.318

LYS 422 - SER 422 : 0.450

TYR 423 - TYR 423 : 0.353

LEU 424 - LEU 424 : 0.625

SER 425 - GLY 425 : 0.808

ALA 426 - VAL 426 : 0.465

GLU 427 - SER 427 : 0.876

GLU 428 - GLU 428 : 1.363

VAL 429 - VAL 429 : 0.718

PRO 430 - PRO 430 : 0.584

ALA 431 - ALA 431 : 1.083

ILE 432 - VAL 432 : 0.646

ASN 433 - HIS 433 : 0.715

ASP 434 - ASP 434 : 0.390

PHE 435 - PHE 435 : 0.378

ASP 436 - ASP 436 : 0.532

ALA 437 - ALA 437 : 0.383

PHE 438 - PHE 438 : 0.311

PHE 439 - PHE 439 : 0.218

PRO 440 - PRO 440 : 0.638

TYR 441 - TYR 441 : 0.669

ASN 442 - ASN 442 : 0.554

ASP 443 - ASP 443 : 0.298

ARG 444 - ARG 444 : 0.375

GLY 445 - GLY 445 : 0.481

ASN 446 - ASN 446 : 0.495

LEU 447 - LEU 447 : 0.443

LEU 448 - LEU 448 : 0.209

ALA 449 - ALA 449 : 0.249

ARG 450 - ARG 450 : 0.229

GLU 451 - ALA 451 : 0.343

GLN 452 - LEU 452 : 0.334

ALA 453 - ALA 453 : 0.325

THR 454 - THR 454 : 0.392

GLY 455 - GLN 455 : 0.325

GLN 456 - GLN 456 : 0.210

ASN 457 - ASN 457 : 0.212

ILE 458 - THR 458 : 0.333

VAL 459 - VAL 459 : 0.616

TRP 460 - TRP 460 : 0.500

GLY 461 - GLY 461 : 0.427

THR 462 - THR 462 : 0.411

GLY 463 - GLY 463 : 0.237

THR 464 - THR 464 : 0.146

HIS 465 - HIS 465 : 0.803

THR 466 - THR 466 : 0.780

HIS 467 - HIS 467 : 0.407

THR 468 - THR 468 : 0.766

PRO 469 - PRO 469 : 0.761

VAL 470 - VAL 470 : 0.493

ASN 471 - ASN 471 : 0.523

VAL 472 - VAL 472 : 0.472

PHE 473 - PHE 473 : 0.557

ALA 474 - ALA 474 : 0.410

TRP 475 - TRP 475 : 0.184

GLY 476 - GLY 476 : 0.221

PRO 477 - PRO 477 : 0.519

ALA 478 - ALA 478 : 0.809

GLU 479 - ASN 479 : 0.530

LYS 480 - ASP 480 : 0.217

ILE 481 - ILE 481 : 0.271

LEU 482 - LEU 482 : 0.184

PRO 483 - PRO 483 : 0.350

VAL 484 - VAL 484 : 0.505

SER 485 - SER 485 : 0.853

LYS 486 - SER 486 : 0.343

ILE 487 - ILE 487 : 0.283

MET 488 - LEU 488 : 0.226

HIS 489 - HIS 489 : 0.191

HIS 490 - HIS 490 : 0.325

SER 491 - SER 491 : 0.358

GLU 492 - GLU 492 : 0.143

LEU 493 - ILE 493 : 0.181

GLY 494 - GLY 494 : 0.164

GLU 495 - GLN 495 : 0.288

TYR 496 - TYR 496 : 0.369

ILE 497 - LEU 497 : 0.378

LYS 498 - LYS 498 : 0.363

GLN 499 - THR 499 : 0.813

GLN 500 - VAL 500 : 0.207

VAL 501 - VAL 501 : 0.404

ASN 502 - ALA 502 : 1.123

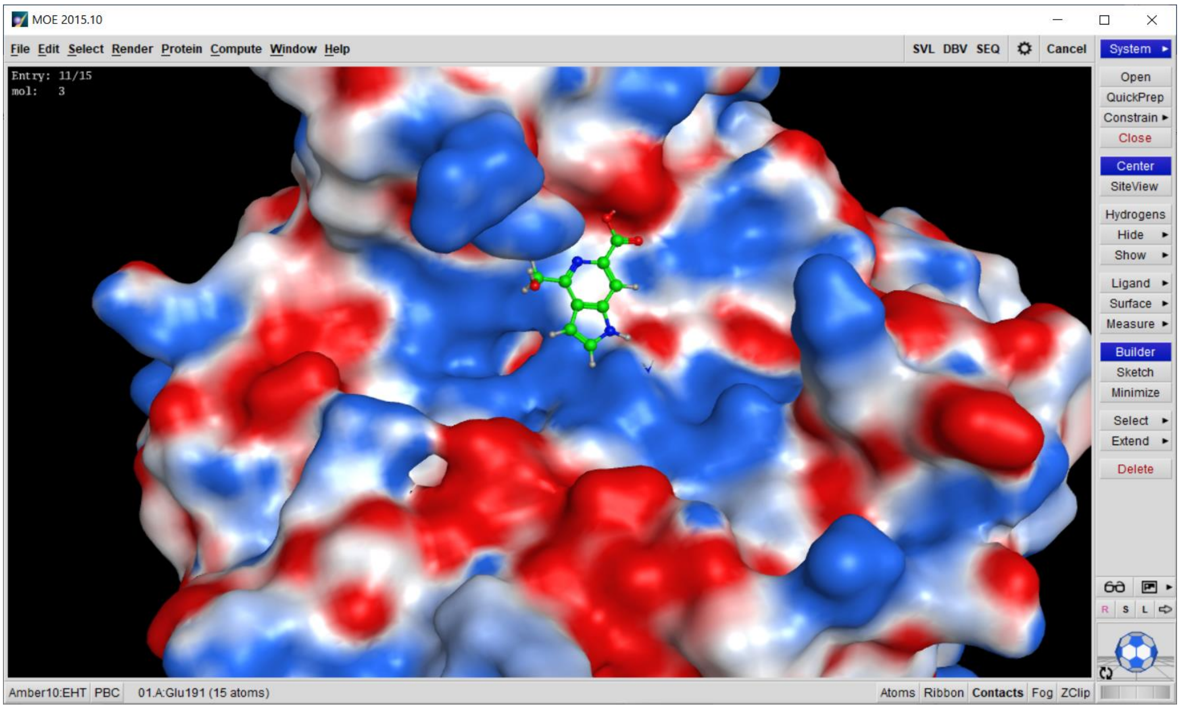

MM:	E	str	ang	oop	tor	vdw	ele
ALL:	-4047.74	292.300	1501.309	23.743	4461.212	-1129.49	-9196.81
SEL:	-164.814	13.856	129.885	1.199	48.343	-21.459	-336.638
INT:	-489.388	0.449	5.838	2.353	119.282	-91.956	-525.354

Figure S4. Electrostatic surface potentials of *Cm*AP entrance to the active site bound with guitarrin C. Positively charged amino acid residues are indicated by blue color. Negatively charged amino acid residues are indicated by red color .

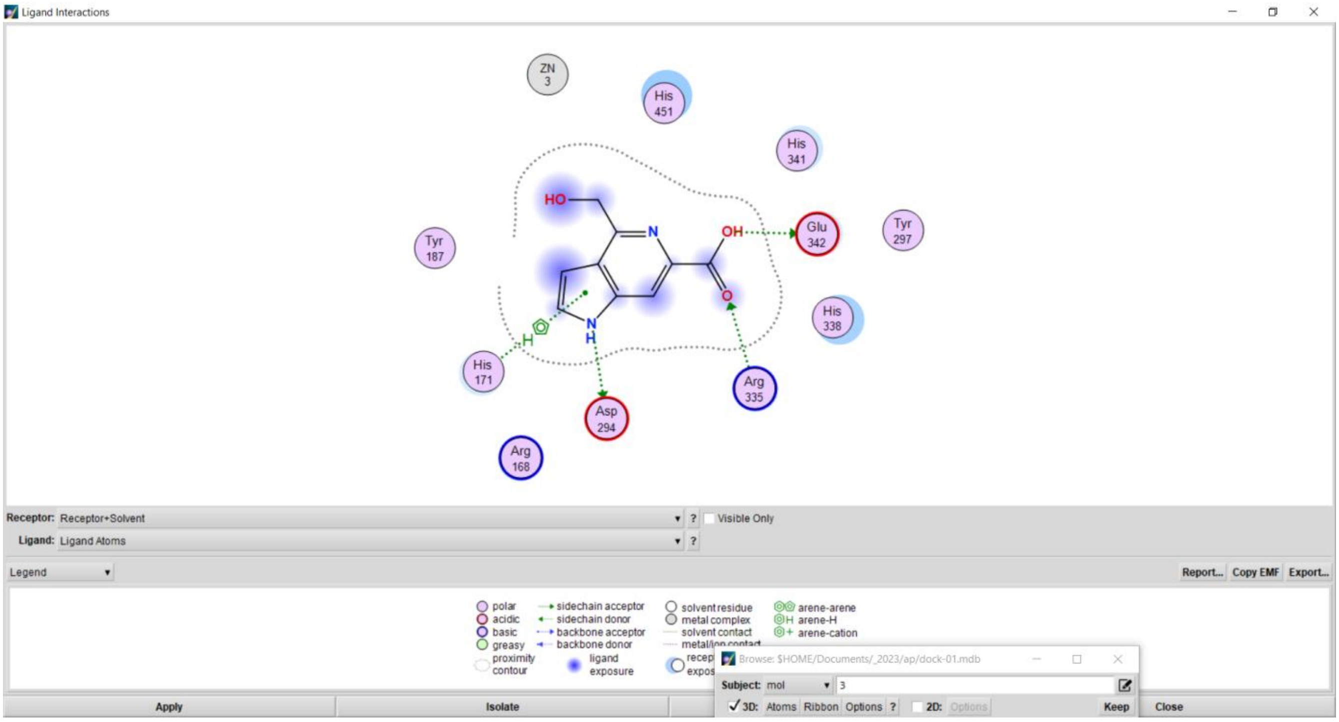

Ligand		Receptor				Interaction	Distance	E (kcal/mol)
N7	7	OD2	ASP	294	(A)	H-donor	2.91	-5.8
O13	13	OE2	GLU	342	(A)	H-donor	2.57	-5.8
O14	14	NH2	ARG	335	(A)	H-acceptor	2.98	-2.7
ZN	3	OD1	ASP	337	(A)	metal	1.72	-1.9
ZN	3	OD1	ASP	337	(A)	ionic	1.72	-22.6
ZN	3	OD2	ASP	337	(A)	ionic	3.07	-4.0
5-ring		NE2	HIS	171	(A)	pi-H	4.00	-2.0

Figure S5. 2-D diagram of the contacts of guitarrin C and human alkaline phosphatase TNAP. The mode of ligand-receptor interactions, the distance and the calculated binding energy are presented in the table below.
